# Towards further defining the proteome of mouse saliva

**DOI:** 10.1186/s12953-015-0068-3

**Published:** 2015-02-25

**Authors:** Anne A Blanchard, Peyman Ezzati, Dmitry Shamshurin, Andreea C Nistor, Etienne Leygue, John A Wilkins, Yvonne Myal

**Affiliations:** Department of Pathology, Faculty of Health Sciences, University of Manitoba, 770 Bannatyne Avenue, Winnipeg, Manitoba Canada; Department of Physiology and Pathophysiology, Faculty of Health Sciences, University of Manitoba, Winnipeg, Canada; Department of Internal Medicine, Faculty of Health Sciences, University of Manitoba, Winnipeg, Canada; Department of Biochemistry and Medical Genetics, Faculty of Health Sciences, University of Manitoba, Winnipeg, Canada

**Keywords:** Mouse/Human saliva, Biomarkers, Mouse salivary proteome, Mass spectrometry, Kallikreins

## Abstract

**Background:**

Knowledge of the mouse salivary proteome is not well documented and as a result, very limited. Currently, several salivary proteins remain unidentified and for some others, their function yet to be determined. The goal of the present study is to utilize mass spectrometry analysis to widen our knowledge of mouse salivary proteins, and through extensive database searches, provide further insight into the array of proteins that can be found in saliva. A comprehensive mouse salivary proteome will also facilitate the development of mouse models to study specific biomarkers of many human diseases.

**Results:**

Individual saliva samples were collected from male and female mice, and later pooled according to sex. Two pools of saliva from female mice (2 samples/pool) and 2 pools of saliva from male mice were used for analysis utilizing high performance liquid chromatograph mass spectrometry (nano-RPLC-MS/MS). The resulting datasets identified 345 proteins: 174 proteins were represented in saliva obtained from both sexes, as well as 82 others that were more female specific and 89 that were more male specific. Of these sex linked proteins, twelve were identified as exclusively sex-limited; 10 unique to males and 2 unique to females. Functional analysis of the 345 proteins identified 128 proteins with catalytic activity characteristics; indicative of proteins involved in digestion, and 35 proteins associated with stress response, host defense, and wound healing functions. Submission of the list of 345 proteins to the BioMart data mining tool in the Ensembl database further allowed us to identify a total of 283 orthologous human genes, of which, 131 proteins were recently reported to be present in the human salivary proteome.

**Conclusions:**

The present study is the most comprehensive list to date of the proteins that constitute the mouse salivary proteome. The data presented can serve as a useful resource for identifying potentially useful biomarkers of human health and disease.

**Electronic supplementary material:**

The online version of this article (doi:10.1186/s12953-015-0068-3) contains supplementary material, which is available to authorized users.

## Background

The oral cavity is a unique environment colonized by bacteria and bathed in salivary fluid. Primarily considered as a component of the digestive process, saliva contains enzymes, including proteases, lipases and glycohydrolases which initiate partial digestion of food components [1]. Many of these enzymes are of bacterial origin and not derived from the salivary glands [1]. Salivary proteins also interact with the oral flora by binding microorganisms, resulting in their aggregation and facilitating their clearance from the oral cavity, serving as receptors for microbial adhesion to host surfaces, possessing antibacterial activity, or serving as microbial nutritional substrates [2]. Thus, saliva and the oral flora are intricately involved in the protection of the oral cavity.

Knowledge of the complexity of saliva in recent years has been expanded such that the term “salivaomics” was coined and is now used to encompass the salivary proteome, transcriptome, microRNA, metabolome and microbiome [3]. These “omes” form the platform for investigating salivary biomarkers for disorders ranging from cancer to infectious disease. The identification of salivary biomarkers of both oral and systemic disease has been a vigorously pursued area of research, primarily attributed to the fact that saliva collection is non-invasive, safe and inexpensive [1,4]. Salivary transcriptome and microbiota biomarkers were recently identified as diagnostic indicators of pancreatic cancer [5], and oral cancer [6] respectively. As well, soluble epidermal growth factor B2 (c-erbB2) has been identified in saliva of women with breast cancer [7]. Therefore salivaomics have shown much promise in cancer biomarker discovery [8].

Although saliva as a proximal biofluid of oral disease is intuitive, the detection of systemic disease is less clear. The mechanisms by which systemic disorders influence the salivome are not well understood. One proposed hypothesis is that blood-derived molecules enter salivary tissues via transcellular or paracellular routes and could potentially influence the molecular constituency of oral fluids [9,10]. More recently, the salivary proteome was shown to be altered through exosomes, microvesicular structures which deliver their contents from distal sites to other parts of the body including the salivary glands [3]. In cancer, it has been demonstrated that tumor-shed exosomes can shuttle proteins and other microbiological components to saliva, thereby altering both the proteome [11] as well as the transcriptome [12] of saliva.

To contribute to the goal of discovery of biomarkers from the salivary proteome, it is important to identify and catalogue the proteins in normal saliva. The human salivary proteome from healthy individuals was recently reported [13,14]. Knowledge of the salivary proteome of other species have not been well documented, with the exception of recent reports of the salivary proteome of goats and sheep [15], the bovine salivary proteome [16], and two recent studies of mouse saliva [17,18]. The most recent analysis of the mouse salivary proteome identified only 81 proteins [17], and therefore, a more in-depth analysis of the mouse salivary proteome is warranted.

The aim of the present study is to provide a more comprehensive listing by utilizing mass spectrometry analysis of the proteins that constitute the mouse salivary proteome, and to provide a reference list necessary for the utilization of mouse models in the study of salivary biomarkers. We report the most comprehensive list to date of proteins which constitute the mouse salivary proteome. Importantly, we also identify those proteins that were found common to both mouse and human. Our study contributes new knowledge to this growing field of proteomics that could also facilitate the diagnosis of human disease in a non-invasive way.

## Results and discussion

### Identification of novel proteins in mouse saliva

**Figure 1:**
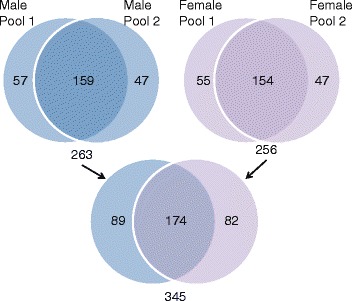
**Venn diagrams of the protein datasets identified in mouse saliva.** Two pools of female mice saliva (Female P1, Female P2) and 2 pools of male saliva (Male P1 and Male P2) were used in generating the Venn Diagram. Following the removal of duplicates, the datasets derived from the Female P1 and Female P2 were then combined, to generate a list of 256 proteins (Additional file 1). Further analysis revealed 154 proteins common to both pools of saliva. Similarly, datasets from the 2 male saliva pools were combined, resulting in a list of 263 proteins (Additional file 2). The compilation of data from Male P1 and Male P2 identified 159 proteins present in both samples. Datasets from the males and females were then combined to identify a total of 345 proteins: 174 proteins confirmed in datasets from both sexes as well as 82 proteins unique to the saliva from females and 89 unique to males (Additional file 3).

To identify the protein composition, saliva samples from both male and female mice were subjected to analysis by mass spectrometry as described in “Methods”. Datasets from each of the 2 pools of saliva from female mice (2 pools, 2 mice per pool) were combined. Following the removal of duplicates, and implementation of a minimum criterion of two unique peptides and a score above −2 a list of 256 proteins was identified (Additional file 1). Analysis of saliva from the male mice resulted in a list of 263 proteins (Additional file 2). After merging the lists of proteins identified in males (263) with the proteins identified in females (256) and removing duplicates, 345 mouse salivary proteins were identified in this study (Additional file 3). Of the 345 proteins, 174 were found in both males and females, 89 were identified only in male saliva, while 82 were female specific (Figure 1). Importantly, these datasets represent the highest number of proteins identified to date from mouse saliva since a recently published list comprised of only 81 proteins [17].

### Delineating protein function through database analysis

Functionally, the majority of proteins identified to date that are found in saliva fall into two categories: digestion and protection [1]. Therefore such findings would not be unexpected among the 345 we identified in this study. To investigate function, proteins were submitted to UniProt gene ontology database analysis [19]. One hundred and twenty-eight proteins were found to have catalytic activity, indicative of proteins involved in digestion. Of these, the largest group was the hydrolases, which include the kallikreins, cathepsins, amylases (Amy1, Amy2), adenosine deaminase (Ada), lactotransferin (Ltf), and chitinase (Chia). Twenty-nine additional proteins were identified as having enzyme regulatory activity components, the largest sub-group being the peptidase inhibitors which included the serpins and whey acidic protein (WAP) four disulfide core domain proteins (Wfdc2, Wfdc12, Wfdc18).

Expectedly, we also identified several proteins that play a role in protection of the teeth and soft tissues of the mouth, and non-immune host defense. It is well recognized that many salivary proteins interact with the microbiota of the oral cavity to promote the colonization of beneficial strains or the clearance of undesirable ones [2,20]. Of particular interest is the prolactin inducible protein, Pip, a protein first identified by us and shown to be highly abundant in both mouse and human saliva [21,22], and was found to be abundant in all 4 saliva sample pools (2 female pools, 2 male pools). We have previously shown that Pip can aggregate oral bacteria [23] inhibiting their activity. Further, using a Pip knockout mouse model we demonstrated that loss of Pip function had significant effects on both the diversity of the oral flora and abundance of specific bacteria of the oral cavity of the Pip null mouse [24]. One hundred and twenty-seven proteins that respond to stimuli were identified; 61 in response to stress stimuli, such as defense response (28 proteins), and wound healing (7 proteins). There was often overlap with regards to protein function and thus many fell into more than one category. Consequently, we combined the defense and wound healing linked proteins into one table which was comprised of 35 proteins (Table 1). Interestingly, the involvement of saliva in wound healing has long been recognized by oral surgeons and indigenous people as well [1].

**Table 1:** **Mouse saliva proteins involved in host defense and wound healing**

**UniProt accession**	**Gene symbol**	**Gene ontology (GO)**
Q14BX6	4933415F23Rik	innate immune response; regulation of phosphorylation
P29699	Ahsg	positive regulation of phagocytosis; regulation of inflammatory response
Q61176	Arg1	response to interleukin-4, lipopolysaccharide, transforming growth factor beta
P01887	B2m	defense response; phagocytic vesicle membrane; positive regulation of T cell mediated cytotoxicity; response to bacteria
P97361	Bpifa1	antibacterial humoral response; innate immune response
Q61114	Bpifb1	innate immune response
P01027	C3	complement activation; inflammatory response; positive regulation of phagocytosis
Q91XA9	Chia	immune system process; positive regulation of chemokine secretion
O35744	Chil3	inflammatory response
P11087	Col1a1	response to transforming growth factor beta stimuli; tooth mineralization; wound healing
O88207	Col5a1	cell adhesion; collagen biosynthetic process; extracellular fibril organization; wound healing
Q9WVJ3	Cpq	carboxypeptidase activity; metallodipeptidase activity; proteolysis; tissue regeneration
P21460	Cst3	cellular response to hydrogen peroxide, axon injury, hypoxia; response to nutrient levels; response to toxic substance
P13020	Gsn	actin filament polymerization; aging; cilium morphogenesis; regulation of cell adhesion; tissue regeneration
Q61646	Hp	antioxidant activity; defense response to bacterium; immune system process; response to hypoxia
O35664	Ifnar2	response to interferon-alpha; type I interferon receptor activity; type I interferon signaling pathway
P01592	Igj	adaptive immune response; antibacterial humoral response; innate immune response
Q9Z1M2	Irgm2	defense response to bacterium; defense response to protozoan; response to interferon-gamma
P00755	Klk1b1	bradykinin biosynthetic process; tissue kallikrein-kinin cascade; vasodilation
P26262	Klkb1	blood coagulation; fibrinolysis; inflammatory response; plasminogen activation;
Q61805	Lbp	defense response to Gram-negative/Gram positive bacterium; innate immune response; regulation of chemokine production
Q5SW46	Lpo	defense response to bacterium; peroxidase activity; response to oxidative stress
P08071	Ltf	antibacterial humoral response; antifungal humoral response; innate immune response in mucosa; positive regulation of toll-like receptor 4 signaling pathway; regulation of cytokine production; regulation of tumor necrosis factor production
P17897	Lyz1	cytolysis; defense response to Gram-negative/ Gram-positive bacterium; lysozyme activity
E9Q5I3	Muc5b	defense response to bacterium; regulation of macrophage activation
Q6LDU8	Ngf	inflammatory response; response to lipopolysaccharide; response to radiation; sensory perception of pain
O08692	Ngp	defense response
O88593	Pglyrp1	defense response to Gram-positive bacterium; innate immune response; negative regulation of inflammatory response; negative regulation of interferon-gamma production; negative regulation of natural killer cell differentiation
P02816	Pip	aspartic-type endopeptidase activity; regulation of immune system process
O09049	Reg3g	MyD88-dependent toll-like receptor signaling pathway; defense response to Gram-positive bacterium; positive regulation of wound healing
P31725	S100a9	chronic inflammatory response; innate immune response; leukocyte chemotaxis; neutrophil aggregation; response to lipopolysaccharide
P07758	Serpina1a	acute-phase response; response to cytokine; response to peptide hormone
Q80YQ1	Thbs1	cellular response to growth factor stimulus; cellular response to tumor necrosis factor; chronic inflammatory response; immune response; inflammatory response
Q9JHY3	Wfdc12	defense response to bacterium; extracellular region; serine-type endopeptidase inhibitor activity
P63101	Ywhaz	histamine secretion by mast cell; response to drug

Submission of mouse salivary proteins to the UniProt gene ontology database identified 35 proteins with functions related to defense and/or wound healing.

Not surprisingly our list consists of several proteins whose functions are yet to be determined. One such protein is the 16.5 kDa submaxillary gland glycoprotein (or salivary protein 1, Spt1) which was first identified by us [25]. Another is Dccp2, which shares homology with members of the common salivary protein-1/Demilune cell and parotid protein (CSP-1/Dccp) family of proteins, suggested to potentially have either antimicrobial or enamel protective activities [26]. Fifty-five other proteins were identified in the mouse saliva that do not have gene ontology annotations in UniProt (Table 2).

**Table 2:** **Mouse salivary proteins with no known gene annotations**

**UniProt**	**Description**
D3YTP1	2310057J18Rik (RIKEN cDNA 2310057 J18, isoform CRA_a)
F7D4R8	5430401F13Rik (Fragment)
E9PWS6	A630073D07Rik
E9QLA8	A830010M20Rik
F6QRE9	BC007180 (Fragment)
Q3UQ05	Bpifb5
A2AJD1	Bpifb9b
E9PYC2	Dcpp2
L7N259	Dcpp3
E9Q0B5	Fcgbp
F6URP1	Gm6619 (Fragment)
A0A075B6A3	Igha
A0A075B5P2	Igkc
A2BHR0	Obp2b
E9PYQ4	Prol1
Q3TUY3	Sbpl (Spermine binding protein-like)
E9QA35	Spata31d1a
D3Z617	Sval2 (Seminal vesicle antigen-like 2, isoform CRA_a)
Q9DA65	16.5 kDa submandibular gland glycoprotein
Q8R1E9	Allergen dI chain C2A (Androgen binding protein beta) (Androgen-binding protein) (Protein Scgb2b27)
Q91WB5	Androgen binding protein alpha (Androgen-binding protein) (Protein Scgb1b27)
Q8JZX1	Androgen binding protein gamma (Protein Scgb2b26) (Salivary androgen-binding protein gamma subunit)
O35176	Androgen-binding protein (Androgen-binding protein eta) (Lacrimal androgen-binding protein eta) (Protein Scgb1b2)
D2XZ31	Androgen-binding protein (Protein Scgb1b29) (Protein Scgb1b7) (Fragment)
Q80XE3	BC051076 protein (Protein BC051076) (Fragment)
P07743	BPI fold-containing family A member 2 (Parotid secretory protein) (PSP)
Q8C1E1	BPI fold-containing family B member 2 (Bactericidal/permeability-increasing protein-like 1)
A2BGH0	BPI fold-containing family B member 4 (Long palate, lung and nasal epithelium carcinoma-associated protein 4)
Q8BU51	BPI fold-containing family B member 6 (Bactericidal/permeability-increasing protein-like 3)
Q9D6P8	Calmodulin-like protein 3
Q99MQ5	Collagen alpha-1(XXV) chain (CLAC-P) [Cleaved into: Collagen-like Alzheimer amyloid plaque component (CLAC)]
Q03401	Cysteine-rich secretory protein 1 (CRISP-1) (Acidic epididymal glycoprotein 1) (Sperm-coating glycoprotein 1) (SCP 1)
Q03402	Cysteine-rich secretory protein 3 (CRISP-3) (Acidic epididymal glycoprotein 2) (Sperm-coating glycoprotein 2) (SCP 2)
P51655	Glypican-4 (K-glypican) [Cleaved into: Secreted glypican-4]
D3YVW2	Golgi integral membrane protein 4
Q8BM72	Heat shock 70 kDa protein 13 (Microsomal stress-70 protein ATPase core)
B1AQ77	Keratin 15, isoform CRA_a (Keratin, type I cytoskeletal 15)
Q3UV17	Keratin, type II cytoskeletal 2 oral (Keratin-76) (K76) (Type-II keratin Kb9)
Q66VB7	Lacrein (Protein Gm1553)
Q14AJ3	Lipocalin 3 (Vomeronasal secretory protein 1)
J3QM75	MCG1039283 (Protein Scgb2b19)
D3YYY1	MCG116526 (Protein Scgb2b7)
G5E8B4	MCG147827 (Secretoglobin family 2B member 2)
G5E8B5	MCG22579 (Secretoglobin family 1C member 1)
Q6GQX2	Nck-associated protein 5-like
Q02819	Nucleobindin-1 (CALNUC)
Q8BHE0	Proline-rich protein 11
Q91X93	Proline-rich protein BstNI subfamily 1 (Protein Prb1)
Q6PDI5	Proteasome-associated protein ECM29 homolog (Ecm29)
Q9CV82	Protein 2310003L06Rik (Fragment)
P97347	Repetin
Q920H1	Secretoglobin family 3A member 2 (Pneumo secretory protein 1) (PnSP-1) (Uteroglobin-related protein 1)
Q6JHY2	Submandibular gland protein C
Q61900	Submaxillary gland androgen-regulated protein 3A (Salivary protein MSG1) (Submaxillary gland androgen-regulated protein 1)
Q6ZPJ0	Testis-expressed sequence 2 protein
Q9CPT4	UPF0556 protein C19orf10 homolog (Interleukin-25) (IL-25) (Stromal cell-derived growth factor SF20)
Q5SXG7	Vitelline membrane outer layer protein 1 homolog

Fifty-seven of the 345 proteins identified in mouse saliva, were found to have no gene ontology attributes in the UniProt database.

### Sex-linked proteins

The small number of mice representing each sex (2 pools; total 4 mice) prohibited statistical analyses and limited conclusions regarding sex-related proteins. Nevertheless, several proteins showed sex-linked quantitative differences. In the present study we identified 82 proteins unique to females and 89 unique to males (Figure 1). However, when a protein was identified in one sex but underrepresented in the other sex by less than 2 peptides, they were not considered as being gender specific. Additionally, proteins that were not represented in both pools from each sex were eliminated. Consequently, a number of proteins were further removed from the gender specific sub-group. Table 3 lists 10 proteins exclusively detected in male saliva and only 2 proteins exclusive to females. Most of these proteins were not highly abundant as indicated by their intensity scores which were not in the top 100 proteins (Additional files 1 and 2). An exception was Klk1b24, a member of the kallikrein-related peptidase family of proteins. Klk1b24 was ranked in the top 40 proteins found in male saliva (Additional file 1), but was not detected in female saliva. The kallikreins and kallikrein-related peptidases are a large family of serine proteases which play important roles in inflammation [27] and have recently been shown to be sex-linked [17]. However, we found that 11 of the 12 kallikrein-related peptidases that were reported as male-specific in the study by Karn et al. [17], were also present in female saliva (Additional file 3). Nonetheless, we detected that, based on the rank and number of unique peptides, 8 of these kallikrein-related peptidases displayed higher levels in males than females, whereas 3 did not show sex-linked quantitative differences in male and females. The levels of Klk1 and Klkb5 were similar in the saliva from both males and females (Additional file 4), in agreement with the observations of Karn et al. [17]. The identification of additional members of the kallikrein-related peptidase family of proteins in female saliva could simply be attributed to an increased depth of analysis, as substantially more proteins were identified in our study. Another plausible explanation could pertain to strain differences; the CD1 strain used in our study versus the C57BL/6 strain used in the Karn et al. study [17].

**Table 3:** **Sex-linked proteins**.

**UniProt**	**Protein name**	**Gene symbol**	**Gene ontology (GO)**
**A) Salivary proteins unique to male mice**			
Q9D6P8	Calmodulin-like protein 3	Calml3	calcium ion binding
Q8BHN3	Neutral alpha-glucosidase AB	Ganab	Golgi apparatus; N-glycan processing; carbohydrate binding; endoplasmic reticulum; glucan 1,3-alpha-glucosidase activity; glucosidase II complex; glucosidase activity; melanosome; protein binding
Q91XA2	Golgi membrane protein 1	Golm1	Golgi apparatus; integral component of membrane; nucleus organization; regulation of lipid metabolic process
Q61754	Kallikrein 1-related peptidase b24	Klk1b24	proteolysis; serine-type endopeptidase activity
Q63836	Selenium-binding protein 2	Selenbp2	cytosol; membrane; nucleus; protein transport; selenium binding
P70277	Alpha-N-acetylgalactosaminide alpha-2,6-sialyltransferase 2	St6galnac2	integral component of Golgi membrane; protein glycosylation; sialyltransferase activity
B1AQJ2	Ubiquitin carboxyl-terminal hydrolase 36	Usp36	cysteine-type peptidase activity; nucleolus; ubiquitin-dependent protein catabolic process
Q80YQ1	Thrombospondin 1	Thbs1	activation of MAPK activity; blood vessel morphogenesis; calcium ion binding; cell adhesion; cell cycle arrest; cell migration; cellular response to growth factor stimulus; cellular response to tumor necrosis factor; (abbreviated)
J3QM75	MCG1039283 (Protein Scgb2b19)	Scgb2b19	extracellular space
A2ANT6	Major urinary protein 6	Mup6	extracellular region; pheromone binding; small molecule binding; transporter activity
**B) Salivary proteins unique to female mice**			
P29699	Alpha-2-HS-glycoprotein	Ahsg	acute-phase response; cysteine-type endopeptidase inhibitor activity; extracellular space; negative regulation of bone mineralization; ossification; positive regulation of phagocytosis; regulation of inflammatory response
Q5GAN1	Angiogenin ribonuclease 5	Ang5	endoribonuclease activity, producing 3'-phosphomonoesters; nucleic acid binding

Ten proteins found in mouse saliva were limited to males and 2 proteins were limited to females. Gene ontologies were derived from UniProt.

### Comparison of the mouse and human salivary proteome

A catalogue of the human salivary proteome from healthy individuals was recently published by Denny et al. [13] identifying over 1100 proteins. This was preceded by a less extensive list published by Wilmarth et al., comprised of 102 proteins [14]. Defining orthologous relationships between two species presents a certain amount of difficulty as many proteins have evolved in different species to several forms and derivatives. For example, the five members of the cystatin family of proteins in the human saliva were represented in the mouse by two members, (gene symbols cst3 and cst10); and lipocalin 1 and lipocalin 2 by four family members in the mouse (gene symbols lcn3, 4, 11, and 14). However, the BioMart data mining tool in the Ensembl genome database (release 76, August 2014 [28]), provides a method of conversion with generally accepted results [29]. We submitted the list of 345 mouse salivary proteins to the BioMart data mining tool which identified 283 orthologous human genes and their corresponding Ensembl gene identification numbers (Additional file 5). Following this analysis, we then compared the mouse proteins with the human proteins identified by Denny et al. [13]. Since the 1166 human salivary proteins, reported by Denny et al. [13] were initially identified by International Protein Index (IPI), a database which has since been deactivated, the DAVID gene identification tool [30,31] was then used to convert the 1166 human protein list to 930 Ensembl gene identification numbers. Of the 283 orthologous genes identified by BioMart from our mouse salivary proteins, 131 were represented in the human salivary proteome (Additional file 5); 93 more than previously reported by Karn et al. [32]. A possible reason for protein differences between the present study and that of the human salivary proteome may be the method of collection (duct versus whole saliva). As well, dietary, hygiene, social and sexual behavior differences may also explain differences in the salivary proteome of the two species. For example, mice use saliva for their fur care, and also lick the fur of other group members for social purposes, or leave traces of saliva, presumably containing pheromone-like substances, to mark their territory. Humans have few remaining functions of saliva in a sociosexual context [33].

## Conclusions

Saliva, as a non-invasive, efficient alternative for the diagnosis of systemic disease holds much promise. In-depth analysis of saliva is important to identify potential biomarkers of disease. Furthermore, such identification in the mouse can facilitate the development of mouse models to study specific biomarkers of many human diseases. The present study has provided the most in-depth analysis of the mouse salivary proteome to date, identifying 345 proteins. Mouse models are commonly used in the study of human diseases and it is well recognized that gender is an important variable that should be considered in many of these models [34-37]. In this study, a search for sex-linked proteins revealed several that were found in male saliva which were absent in females. Additionally, comparison of the mouse proteins with the recently published most extensive catalogue to date, of the human salivary proteome from healthy individuals [13], has allowed the identification of 131 proteins present in both human and mouse saliva. These studies therefore contribute further insight into the growing number of proteins that may be potentially useful biomarkers of health and disease.

## Methods

### Saliva collection

Mice were housed in the animal care facility at the University of Manitoba. All animal protocols were approved by the University of Manitoba Animal Care and Use Committee in accordance with the Canadian Council for Animal Care Guidelines. Saliva was collected at 7–8 weeks of age from 4 female and 4 male CD1 mice. Briefly, animals were anesthetized in a chamber infused with 4% isofluorane, and salivation was induced by subcutaneous administration of 10 mg/kg pilocarpine (Sigma-Aldrich Canada Co.Oakville, ON, Canada) in phosphate buffered saline. Saliva was collected with a pipet over a 15 min period and transferred to a microcentrifuge tube containing protease inhibitor (Complete Mini, Roche Diagnostics, Laval, PQ, Canada). The saliva was then vortexed for 1 min, centrifuged at 16000 g for 5 min at 4°C, transferred to a new tube, leaving behind any precipitated debris from the mouth, and then stored at −80°C. This protocol excludes intact microorganisms present in the collected saliva from subsequently being processed with the salivary proteins.

### Sample preparation

The protein concentration of each saliva sample was determined using the Pierce BCA assay kit (Fisher Scientific). Saliva samples collected from two female mice were combined in equal protein amounts to form a pooled sample (Female P1), and similarly pooled from the two other female mice (Female P2). Likewise, saliva collected from 4 male mice was combined into 2 pools (Male P1 and Male P2). Proteomic analysis was then carried out at the Manitoba Centre for Proteomics and Systems Biology at the University of Manitoba.

### Protein digestion and peptide purification

A modified version of the filter aided sample preparation protocol [38] was used. Proteins from each pellet pool were resuspended in 1 ml of lysis buffer (1% [wt/vol] sodium dodecyl sulfate, 100 mM Tris–HCl, 0.1 M dithiothreitol [pH 7.6]) and heated at 95°C for 5 min and subsequently centrifuged at 16,000 × *g* for 20 min. The resulting supernatant was transferred to an Amicon Ultra-15 10 K filter device (Millipore, Billerica, MA) and washed three times in 12 ml of urea solution (8 M urea in 0.1 M Tris–HCl [pH 8.5]). Each wash step included centrifugation at 4,000 × *g* for at least 10 min until the final volume remaining in the filter tube was <0.5 ml. 100 mM -iodoacetamide solution (in 8 M urea solution) was added to the filter device, and left at room temperature in the dark for 20 min. After centrifugation, the filter membrane was washed twice with an additional 12 ml of urea solution. A 50-μl aliquot was taken from the filter unit and analyzed by using a BCA protein assay kit (Pierce Chemical Co., Rockford, IL) to estimate the total protein content of the sample. The filter membrane was washed twice with 12 ml of 50 mM ammonium bicarbonate in water, and the remaining protein was trypsin digested for 18 h at room temperature (trypsin/protein ratio, 1 μg:100 μg). On the following day, the filter unit was transferred to a new collection tube and spun at 4,000 × *g* for 10 min, and the filtrate was retained for downstream analysis. The membrane was washed with 1 ml of 0.5 M NaCl, and the resulting filtrate was combined with the corresponding previous filtrate and stored at −80°C and dried in speed vac. Dried peptides were resuspended in 0.1% TFA and desalted by 100-mm C_18_ column (5-μm Luna C18) [39]; Phenomenex, Torrance, CA) and eluted using 80% (vol/vol) acetonitrile. Purified aliquots were lyophilized and redissolved in buffer A (0.1% formic acid in water). Peptide concentrations in the combined filtrate were measured using a NanoDrop spectrophotometer (Thermo Fisher Scientific Inc, MA,USA) for subsequent mass spectrometry analysis.

### Proteomic analysis by nano-RPLC-MS/MS

Saliva samples were analyzed by nano-RPLC-MS/MS using an A splitless Ultra 2D Plus [Eksigent, Dublin, CA] system coupled to a high speed Triple TOF™ 5600 mass spectrometer [AB SCIEX, Concord, Canada] as described previously [38-42] ~ 3ug peptides from each pool were injected via a PepMap100 trap column [0.3 × 5 mm, 5 μm, 100 Å, Dionex, Sunnyvale, CA], and a 100 μm × 150 mm analytical column packed with 5 μm Luna C18(2) was used prior to MS/MS analysis. Both eluents A (water) and B (99% acetonitrile) contained 0.1% formic acid as an ion-pairing modifier. The tryptic digest was analyzed with 180 minutes gradient. Eluent B had a gradient from 0% to 35% over 165 minutes, 35% to 85% in 1 minute and was kept at 85% for 5 minutes at a flow rate of 500 nL/min. Key parameter settings for the TripleTOF 5600 mass spectrometer were as follows: ionspray voltage floating (ISVF) 3000 V, curtain gas (CUR) 25, interface heater temperature (IHT) 150, ion source gas 1 (GS1) 25, declustering potential (DP) 80 V. All data was acquired using information-dependent acquisition (IDA) mode with Analyst TF 1.5 software [AB SCIEX, USA]. For IDA parameters, 0.25 s MS survey scan in the mass range of 400–1250 were followed by 20 MS/MS scans of 100 ms in the mass range of 100–1600 (total cycle time: 2.3 s). Switching criteria were set to ions greater than mass to charge ratio (m/z) 400 and smaller than m/z 1250 with a charge state of 2–5 and an abundance threshold of more than 150 counts. Former target ions were excluded for 5 seconds. A sweeping collision energy setting of 37 ± 15 eV was applied to all precursor ions for collision-induced dissociation.

### Software analysis of protein identification and function

Spectra files were generated using Analyst® TF 1.6.2 Software and converted into mascot generic file format (.mgf) using AB SCIEX MS Data converter [AB SCIEX, Foster City, CA]. These files containing the MS/MS spectra information were submitted for protein identification by the X!Tandem GPM (http://www.thegpm.org). The following parameters were used: (i) enzyme, trypsin; (ii) one missed cleavage allowed; (iii) fixed modification, carbamidomethylation of cysteines (iv) variable modification, oxidation of methionine; (v) peptide tolerance, 20 ppm; and (vi) MS/MS tolerance, 20 ppm. LC-MS/MS data of were searched against the Mus musculus proteome. Any proteins identified as belonging to any species other than mouse were eliminated from our list.

## Additional files

Additional file 1:
**Proteins identified in saliva from female mice.** 256 proteins were identified by 2 or more unique peptides. Additional file 2:
**Proteins identified in saliva from male mice.** 263 proteins were identified by 2 or more unique peptides. Additional file 3:
**Proteins identified in mouse saliva.** 345 proteins were identified by 2 or more unique peptides. Additional file 4:
**Comparative levels of kallikreins in saliva from male and female mice.**
Additional file 5:
**Comparison between human and mouse salivary proteomes.** 283 human genes orthologous to the mouse salivary proteins and their corresponding Ensembl gene identification numbers are shown. This list was aligned with the human salivary proteome [13], and 131 proteins were found common to both mouse and human saliva.

## Abbreviations

2D-LCMS: Two dimensional liquid chromatography\mass spectrometry
DTT: Dithiothreitol
IAA: Iodoacetamide
KO: Knockout
MS/MS: Tandem mass spectrometry
Pip: Prolactin inducible protein
TBS-T: Tris-buffered saline with 0.05% Tween 20
TFA: Trifluoroacetic acid
WT: Wild-type
